# Extraocular Sebaceous Carcinoma Presenting as a Presumed Sebaceous Cyst in a Patient With Lynch Syndrome

**DOI:** 10.7759/cureus.117291

**Published:** 2026-10-01

**Authors:** Amber Hands, Jennifer Burgoyne

**Affiliations:** 1 Department of Surgery, University of Kansas School of Medicine, Wichita, USA

**Keywords:** lynch syndrome phenotype, mismatch repair genes, muir-torre syndrome, sebaceous carcinoma, sebaceous neoplasm

## Abstract

Extraocular sebaceous carcinoma is a rare cutaneous malignancy that may clinically mimic benign lesions, potentially delaying diagnosis. It is associated with Muir-Torre syndrome, a phenotypic variant of Lynch syndrome characterized by sebaceous neoplasms and internal malignancies. We report a 60-year-old man with germline-confirmed Lynch syndrome and a history of resected urothelial carcinoma who presented with a painless, progressively enlarging right upper cheek mass that had been present for six months. Examination revealed a firm, freely mobile, 15 × 12 mm subcutaneous nodule without overlying skin changes or regional lymphadenopathy. Biopsy demonstrated well-to-moderately differentiated sebaceous carcinoma with invasion into skeletal muscle. Immunohistochemical evaluation demonstrated loss of MSH2 and MSH6 expression, consistent with mismatch repair deficiency and the patient's known germline Lynch syndrome. Given muscle invasion, the patient underwent re-excision with intraoperative frozen-section analysis, which showed no residual malignancy, and final pathology confirmed clear margins. Postoperative PET scan showed no evidence of metastatic disease. At four months, the patient remains recurrence-free and continues surveillance for local recurrence and other Lynch syndrome-associated malignancies. This case highlights the importance of maintaining a high index of suspicion for sebaceous carcinoma in patients with Lynch syndrome, even when lesions present as clinically benign-appearing subcutaneous nodules. Early biopsy, complete surgical excision, and appropriate systemic surveillance are essential for management and detection of associated malignancies.

## Introduction

Lynch syndrome is an autosomal dominant hereditary cancer predisposition syndrome caused by pathogenic germline variants in the mismatch repair (MMR) genes mutL homolog 1 (MLH1), mutS homolog 2 (MSH2), mutS homolog 6 (MSH6), and PMS1 homolog 2 (PMS2), or by epithelial cell adhesion molecule (EPCAM) deletions that silence MSH2 [1]. Impaired MMR increases the risk of colorectal, endometrial, urothelial, and several other malignancies [1]. Muir-Torre syndrome (MTS) is a less common phenotypic variant within the Lynch syndrome spectrum, defined by the presence of sebaceous neoplasms together with internal malignancies [2,3]. Sebaceous neoplasms are clinically relevant in this setting because they may be the first manifestation of the syndrome and should prompt MMR testing and systemic surveillance. Sebaceous carcinoma is an uncommon but aggressive cutaneous malignancy that may serve as a clinical marker of MTS [4-6].

We report a patient with known germline Lynch syndrome who developed extraocular sebaceous carcinoma of the right upper cheek without evidence of regional or distant metastasis. This case is notable because the tumor presented as a freely mobile subcutaneous nodule in a common location for benign pathologies, closely mimicking a benign lesion. It highlights the need for a high index of suspicion for malignant cutaneous lesions in patients with Lynch syndrome, as enlarging nodules should not be presumed benign and warrant prompt biopsy and histopathologic evaluation.

## Case presentation

A 60-year-old man with Lynch syndrome presented with a painless, enlarging mass of the right upper cheek. Lynch syndrome had been diagnosed nine years prior to presentation following genetic evaluation prompted by a family history of bladder and gastrointestinal malignancies. Four years before the current presentation, he was diagnosed with urothelial carcinoma, which was treated with surgical resection. He has since undergone annual colonoscopic surveillance, with unremarkable findings, and has had no evidence of recurrent malignancy. The specific germline variant and further details of his urothelial carcinoma, including tumor site, stage, and grade, were not available in the records reviewed. The cheek lesion was first noticed six months before presentation and had progressively increased in size, eventually interfering with his peripheral vision. He denied pain, drainage, bleeding, pruritus, or ulceration.

On examination, a firm, freely mobile, subcutaneous nodule measuring 15 × 12 mm was present over the right upper cheek without overlying skin changes (Figure 1). Best-corrected visual acuity was 20/20 in both eyes. Slit-lamp examination demonstrated 1+ bilateral nuclear sclerotic cataracts, with an otherwise unremarkable anterior segment examination. There was no evidence of ocular involvement or regional lymphadenopathy. The differential diagnosis included an epidermal inclusion cyst (sebaceous cyst), basal cell carcinoma, intradermal nevus, sebaceous hyperplasia, sebaceous carcinoma, and keratoacanthoma. Given the enlarging nature and uncertain diagnosis of the lesion, an incisional biopsy was performed at the right upper cheek.

**Figure 1:**
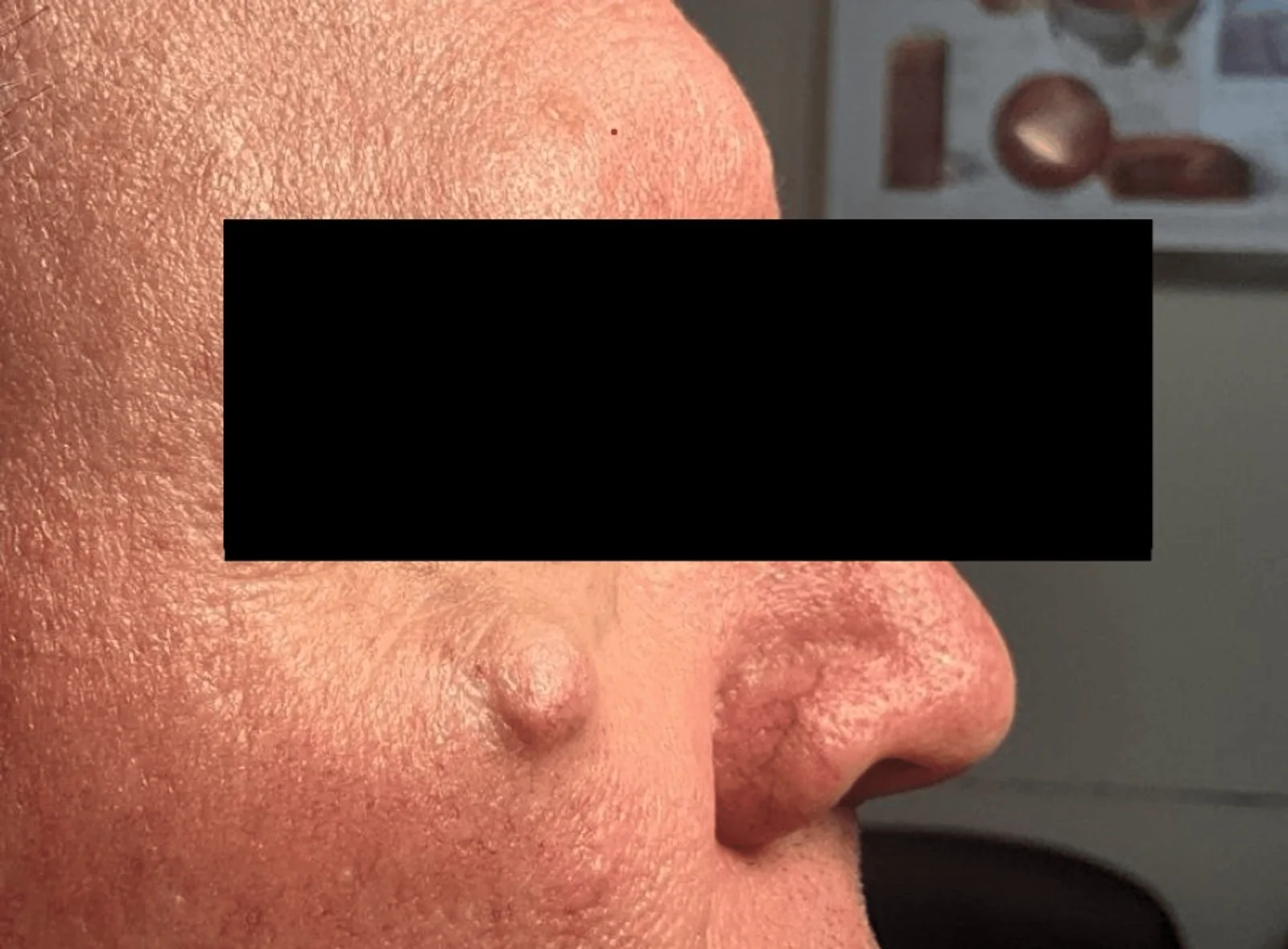
Preoperative neoplasm of unknown origin at the time of the biopsy

Histopathologic examination of the 3.0 × 1.0 cm incisional biopsy specimen demonstrated a well- to moderately differentiated sebaceous carcinoma measuring 9.5 × 7.5 mm in greatest dimension with invasion into the underlying skeletal muscle. Surgical margins were positive for tumor involvement at the 3 o'clock position, as oriented on the specimen. Hematoxylin and eosin (H&E) staining demonstrated an infiltrative neoplasm composed of atypical sebaceous cells arranged in a lobular architecture, with prominent cytologic atypia and abundant vacuolated/foamy cytoplasm consistent with sebaceous differentiation (Figure 2). At lower magnification (40×), the infiltrative nature of the tumor and its extension into the surrounding tissue are demonstrated (Figure 3). Immunohistochemical evaluation of the MMR proteins MLH1, MSH2, MSH6, and PMS2 was performed. Loss of MSH2 and MSH6 expression was identified, with retained MLH1 and PMS2, consistent with MMR deficiency and in keeping with his known germline Lynch syndrome.

**Figure 2:**
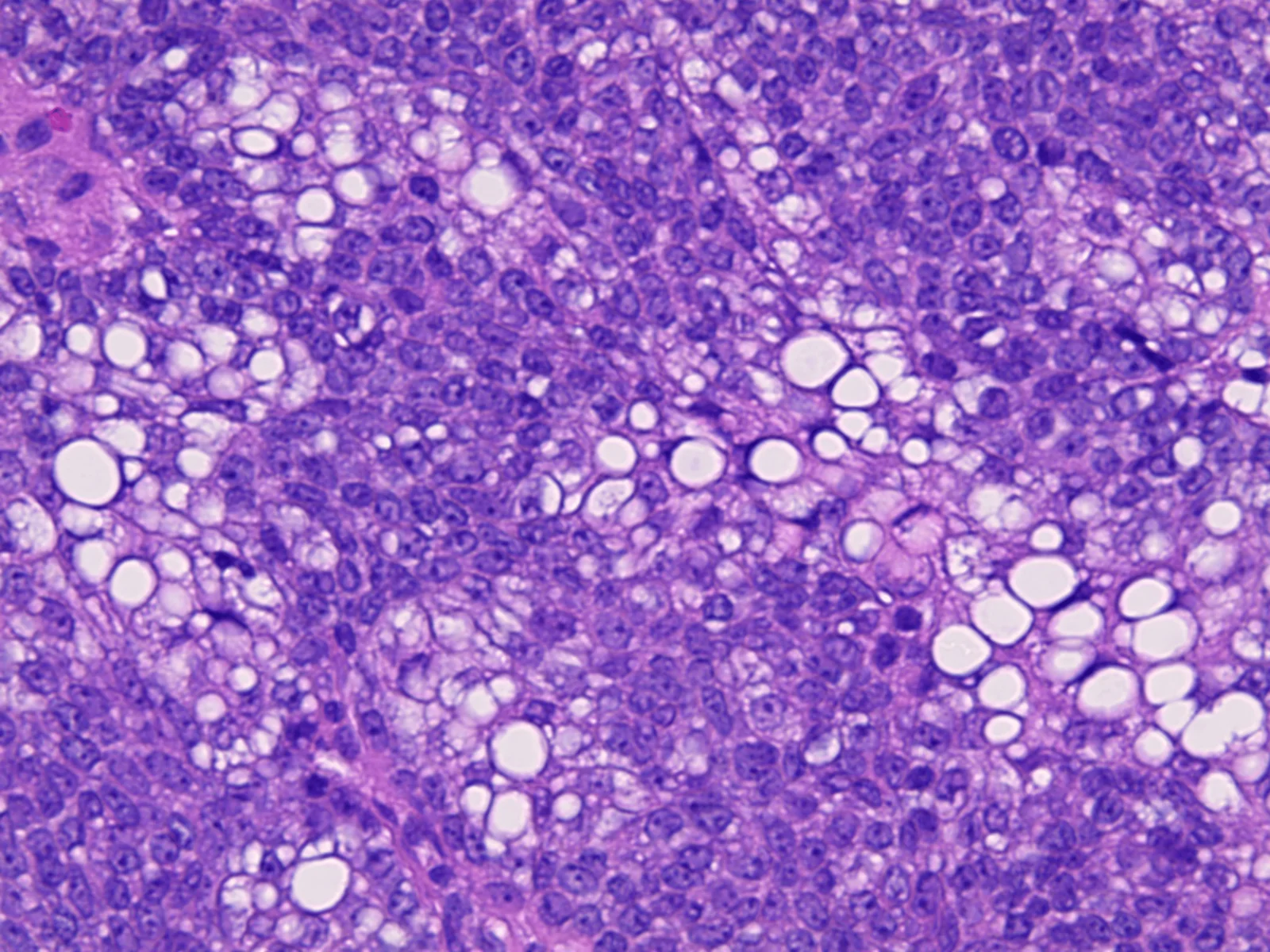
High-power H&E staining (400×) demonstrating sebaceous carcinoma of the right upper cheek

**Figure 3:**
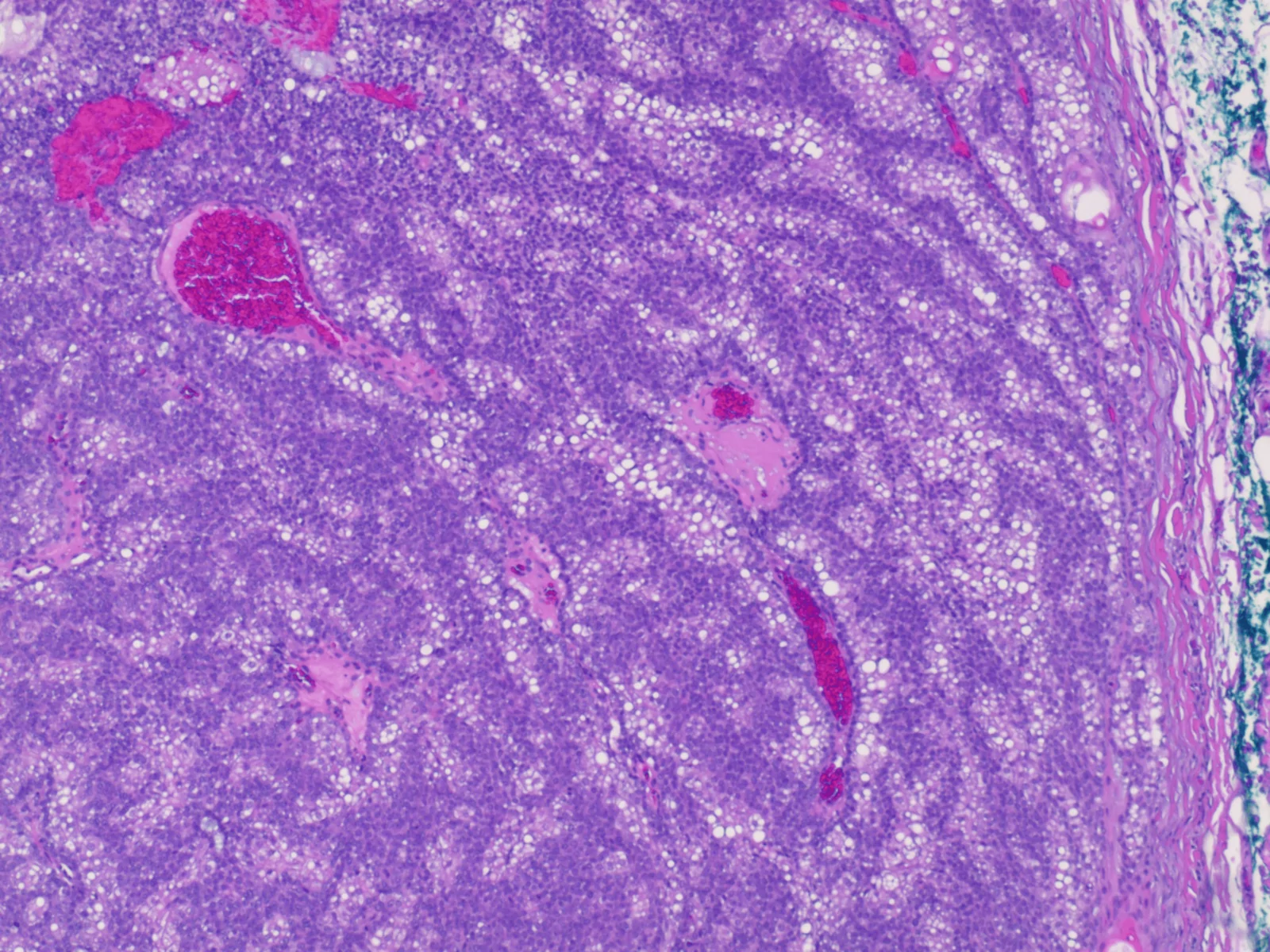
H&E staining (40x) demonstrating infiltrative nature of sebaceous carcinoma with cellular atypia

Following the initial diagnosis, and given the positive margin and muscle invasion, the patient underwent planned re-excision with intraoperative frozen-section margin assessment. Evaluation of the re-excision margin corresponding to the previously involved 3 o'clock position (as oriented on the specimen) demonstrated fibroadipose tissue with chronic inflammation and granulomatous reaction without evidence of residual malignancy. Final pathology confirmed complete excision. A postoperative PET scan demonstrated no evidence of regional or distant metastatic disease. At four-month follow-up, the patient remained free of local recurrence and continues surveillance for recurrence and other Lynch syndrome-associated malignancies.

## Discussion

Lynch syndrome is an autosomal dominant hereditary cancer predisposition syndrome that affects approximately one in 300 individuals [1]. It is caused by pathogenic variants in the DNA MMR genes MLH1, MSH2, MSH6, and PMS2, or by deletions of EPCAM, and is associated with an increased lifetime risk of colorectal, endometrial, ovarian, gastric, urothelial, and other visceral malignancies, including sebaceous neoplasms [1]. Because of this elevated cancer risk, patients with Lynch syndrome require lifelong surveillance, including routine colonoscopy, upper endoscopy, dermatologic examinations, and urologic monitoring to facilitate early detection and treatment of associated malignancies [1].

Muir-Torre syndrome (MTS) is a phenotypic variant of Lynch syndrome characterized by sebaceous neoplasms and internal malignancies resulting from germline MMR gene mutations [2,3]. MTS is present in approximately 9.2% of patients with Lynch syndrome and occurs predominantly in males and Caucasians [4]. Cutaneous manifestations include sebaceous adenomas, sebaceous carcinomas, and keratoacanthomas [4].

Sebaceous carcinoma is the third most common eyelid malignancy after basal cell carcinoma and squamous cell carcinoma. It has an estimated incidence of one to two cases per million individuals and predominantly affects men between 60 and 79 years of age [4]. Because of its variable clinical appearance, sebaceous carcinoma is frequently mistaken for benign lesions, including epidermal inclusion cysts, sebaceous hyperplasia, chalazion, or basal cell carcinoma, often resulting in delayed diagnosis [7-9]. It can present as an extraocular lesion of the head and neck, typically as an ulcerated yellow nodule [8], although it has also been reported in the parotid gland, prostate, breast, and ovary. Histopathologic examination remains the gold standard for diagnosis because the clinical appearance alone is insufficient to reliably distinguish sebaceous carcinoma from other benign or malignant cutaneous lesions [4,5]. In our patient, a history of Lynch syndrome maintained a high clinical suspicion for MTS despite the lesion's benign appearance. An incisional biopsy demonstrated a well- to moderately differentiated sebaceous carcinoma with skeletal muscle invasion. Well-differentiated sebaceous carcinomas characteristically demonstrate sebaceous differentiation with foamy cytoplasm and may exhibit comedo-type necrosis and pagetoid spread [4]. Histopathologically, sebaceous carcinomas are classified into four subtypes: papillary, lobular, comedocarcinoma, and mixed [4].

Complete surgical excision remains the standard treatment for sebaceous carcinoma, with radiation therapy reserved for patients with lymph node involvement or unresectable disease [4,6,10]. Reported recurrence rates approach 30%, with a five-year survival of approximately 78% for localized disease compared with 50% for metastatic disease [9]. Sebaceous carcinomas without MMR mutations have also been reported; risk factors include ultraviolet (UV) radiation exposure, immunosuppression, and increasing age [10,11].

## Conclusions

This case highlights the importance of maintaining a high index of suspicion for sebaceous carcinoma in patients with Lynch syndrome who develop new or enlarging cutaneous or subcutaneous nodules, even when the lesion is freely mobile and resembles a benign cyst. Prompt biopsy allows timely diagnosis and definitive surgical management, and should be followed by appropriate surveillance for metastatic disease and other Lynch syndrome-associated malignancies. Limitations of this report include the short follow-up (four months) and the fact that the germline variant from the patient's prior genetic diagnosis was not available, although MMR immunohistochemistry was performed on the tumor.
